# Complement C1q and von Willebrand factor interaction in atherosclerosis of human carotid artery

**DOI:** 10.3389/fimmu.2023.1265387

**Published:** 2023-12-14

**Authors:** Kristina Schulz, Claudia Donat, Mukesh Punjabi, Katharina Glatz, Beat Kaufmann, Marten Trendelenburg

**Affiliations:** ^1^ Laboratory of Clinical Immunology, Department of Biomedicine, University of Basel, Basel, Switzerland; ^2^ Division of Internal Medicine, University Hospital of Basel, Basel, Switzerland; ^3^ Laboratory of Cardiovascular Molecular Imaging, Department of Biomedicine, University of Basel, Basel, Switzerland; ^4^ Institute for Pathology, University Hospital of Basel, Basel, Switzerland; ^5^ Division of Cardiology, University Hospital of Basel, Basel, Switzerland

**Keywords:** atherosclerosis, complement, C1q, von Willebrand factor, hemostasis, vWF

## Abstract

Atherosclerosis is an inflammatory disease of the vessel wall, with cholesterol crystal (CC) deposition being a hallmark of the disease. As evidence for a cross-talk between complement activation and hemostasis on CC surfaces has been limited to *in vitro* data, the aim of this study was to demonstrate the presence of C1q-vWF complexes in human atherosclerosis *ex vivo*. We used immunofluorescence staining and a proximity ligation assay (PLA, Duolink^®^) to examine the presence, localization, and co-localization of C1q and vWF in frozen sections of human carotid arteries with atherosclerosis or without atherosclerotic changes as well as material from thrombendarteriectomy. We observed significantly higher levels of C1q and vWF in healthy tissue compared to diseased material and greater co-localization in the PLA in healthy samples than in diseased samples. In diseased samples, fluorescence signals were highest in locations encompassing atheroma and foam cells. While there was overall reduced signal in areas with CCs, the staining was spotty, and there was evidence of co-localization on individual CCs. Thus, we demonstrate the presence of C1q-vWF complexes in human carotid arteries *ex vivo*, which was most abundant in healthy endothelial and subendothelial space and reduced in diseased tissue. C1q-vWF interaction can also be demonstrated on the CC surface.

## 1 Introduction

Atherosclerosis is a highly prevalent cardiovascular disease, accounting for high morbidity and mortality and consequently presenting a massive health burden (1). A hallmark of atherosclerosis is the presence of cholesterol crystals (CCs) in the intima of arteries from early lesions to late plaques. CCs trigger inflammation of the arterial wall, involving the complement system as a pivotal part of the innate immune system (2–4). C1q, the initiation molecule of the classical complement pathway, enhances the clearance of apoptotic cells (5, 6), atherogenic lipoproteins (7), and CCs by phagocytes (3). While there is additional evidence for atheroprotective effects of C1q in early atherosclerosis *in vivo* (8, 9), the involvement of the downstream complement cascade after binding to oxidized low-density lipoproteins or CCs can in particular drive the progression of atherosclerosis in animal models (10, 11). Thus, the role of C1q in atherosclerosis remains to be determined and appears to be controversial.

Von Willebrand factor (vWF), the starter molecule of the primary hemostasis, is centrally involved in primary clot formation and bleeding arrest by connecting platelets firmly to subendothelial tissue (12). Thrombus formation upon plaque rupture can cause most severe symptomatic conditions of atherosclerosis, such as myocardial infarction or stroke. So far, neither an indisputable protective nor harmful contribution to atherogenesis has been proven for vWF (13).

There is accumulating evidence for a cross-talk between complement and coagulation on multiple levels (14, 15), such as CC-induced complement-dependent activation of hemostasis (16). C1q-vWF interaction has also been shown to modulate inflammatory responses as bound C1q decreases the release of pro-inflammatory cytokines by phagocytes (17), and this effect is even enhanced by the presence of vWF being bound to C1q using CCs as a carrier (18). While the presence of C1q-vWF complexes has been described in the glomeruli of SLE patients with proliferative lupus nephritis (19), to date, C1q-vWF complexes have not been studied in atherosclerosis. Therefore, this study aimed at demonstrating the presence of C1q-vWF complexes in human atherosclerosis *ex vivo*.

## 2 Materials and methods

### 2.1 Immunofluorescence, trichrome staining, and PLA of human carotid arteries

#### 2.1.1 Ethical approval

The collection and use of patient tissue were approved by the local Ethical Committee of Northwestern and Central Switzerland (EKNZ No. 2019-01490).

#### 2.1.2 Patients and processing of samples

Carotid artery plaque tissue was obtained from seven patients who underwent a clinically indicated thromboendarterectomy procedure at the Department of Vascular Surgery of the University Hospital Basel, Switzerland (TE, n=7) (20). An internal carotid artery segment was taken as a control from seven autopsied patients with low-grade or absent atherosclerosis at the Department of Pathology of the University of Basel (healthy autopsy material, HA, n=7). Similarly, a second group with diseased material was generated from seven autopsied patients with atherosclerotic manifestation in the carotids (diseased autopsy material, DA, n=7). Patient characteristics regarding co-morbidities and medication are summarized in **Table 1**. In order to obtain specimens, all individuals or their relatives gave written informed consent prior to participation in the study (TE specimens) or for research on deceased persons (HA and DA specimens), respectively. Immediately after the procedure, specimens were embedded in optimal cutting temperature (OCT) media (CellPath) and snap-frozen in liquid nitrogen. The specimens were then cut with a cryostat in 6μm sections and laid onto SuperFrost Plus slides (Menzel, Germany).

**Table 1:** Patient characteristics.

	Age	Sex	Symptomatic	Hypertension	Diabetes	Smoking	BMI kg/m²	Cause of death	CRP mg/l	Statin	Antiplatelet Agent
Healthy autopsied*	76	F		No	No	No	25.1	Septic shock by cholangitis	201	No	No
	82	M	NA	Yes	Yes	NA	34.1	MI	435	Yes	Yes
	62	M	NA	No	No	No	27.9	PE	77.8	Yes	No
	71	M	NA	Yes	No	St.p.	20.6	PML	21.2	Yes	Yes
	73	M	NA	NA	NA	NA	24.3	MI	NA	NA	NA
	87	M	NA	NA	NA	NA	27.5	TBI	1.8	NA	NA
	83	M	NA	Yes	No	NA	NA	Sepsis by enterocolitis	293	No	No
Diseased autopsied*	66	F	No	Yes	No	Yes	39.9	Ruptured Aorta	125	No	No
	80	F	No	Yes	No	NA	19.7	COPD exacerbation	104	No	No
	69	M	No	Yes	No	NA	22.2	Aspiration pneumonia	340	No	No
	95	M	No	Yes	Yes	St.p.	27	MOF by sepsis	174	No	No
	76	F	No	Yes	Yes	No	NA	MI	139	No	No
	86	F	No	Yes	No	No	24.4	Native valve endocarditis	92	Yes	No
	70	M	No	Yes	Yes	Yes	28.5	Metastatic tumor disease	159	Yes	Yes
Diseased TE**	46	M	Yes	Yes	No	NA	20.9	NA	0.5	Yes	Yes
	75	F	No	Yes	No	Yes	23.0	NA	0.5	Yes	Yes
	85	M	No	Yes	Yes	St.p.	NA	NA	1.3	Yes	Yes
	73	F	Yes	Yes	Yes	Yes	NA	NA	4.9	Yes	Yes
	84	M	No	Yes	Yes	No	28.1	NA	1.2	Yes	No
	77	F	Yes	Yes	Yes	No	23.9	NA	1.6	Yes	Yes
	64	F	Yes	Yes	Yes	NA	NA	NA	3.8	Yes	Yes

Patient characteristics of specimens used in the study are provided. *Provider (source) of control specimen is Division of Pathology of University Hospital Basel, Switzerland. **Provider (source) of diseased specimen obtained by thromboendarterectomy is Division of Cardiology, University Hospital Basel, Switzerland; BMI, body mass index; COPD, chronic obstructive pulmonary disease; MI, myocardial infarction; MOF, multiorgan failure; NA, not applicable or available; PE, pulmonary embolism; PML, progressive multifocal leukoencephalopathy; St.p., status post; TBI, traumatic brain injury.

#### 2.1.3 Immunofluorescence

Sections were thawed for 30min, washed with PBS (Life Technology, Carlsbad, CA, USA)/0.05% Tween (MiliporeSigma, St. Louis, MO, USA), and blocked with PBS/0.05% Tween/1% BSA (MiliporeSigma)/1% FCS (Life Technology) (PBSTBF) for 30min. Sections were then incubated with the following primary monoclonal antibodies: rabbit anti-human vWF (Abcam, Cambridge, UK) at a concentration of 25μg/ml and in-house mouse anti-human C1q cell culture supernatant (clone 32A6) at a 1:5 dilution, as well as clone 23D11 and 12F10 in a subgroup (21). While clone 23D11 and clone 12F10 were used to confirm C1q distribution, main experiments were performed with clone 32A6 due to its better staining properties. Isotype control antibodies were mouse anti-IgG (Southern Biotech) and rabbit anti-IgG (Southern Biotech, Birmingham, USA), as well as mouse anti-IgG-kappa (Invitrogen, Carlsbad, CA, USA) in a subgroup, in PBSTBF for 1h. Concentrations of isotype control antibodies were adapted to match the primary antibody concentration, which was estimated by nanodrop and determination of the concentration of the purified in-house anti-C1q antibody. Afterwards, sections were incubated with the secondary antibodies goat anti-mouse IgG-AF750 (Invitrogen, Carlsbad, CA, USA) at a concentration of 10μg/ml and goat anti-rabbit IgG-AF647 (Jackson, Cambrigeshire, UK) at a concentration of 2.5μg/ml for 30min in the dark. Finally, sections were washed with PBS/0.05% Tween three times and with distilled water once before mounting with Fluoroshield (MiliporeSigma). All steps were performed at RT. Afterwards, specimens were stored in the dark at 4°C until microscopy analysis. In addition, serial sections were anatomically stained using standard trichrome stain.

In a subset of samples (n=12), we performed additional C3c and C4d stains. For the C3c stain the protocol was applied as described above using rabbit anti-C3c antibody (Dako) as the primary antibody at a concentration of 12 μg/ml and goat anti-rabbit IgG-AF647 (Jackson, Cambrigeshire, UK) at a concentration of 2.5μg/ml. The C4d stains were performed at the Department of Pathology of the University of Basel according to the standardized staining procedure for clinical diagnostics. Briefly, sections were washed with cold PBS/0.05% Tween for 5 min. Sections were then incubated 30 min at RT with polyclonal rabbit anti-C4d (BI-RC4D, Biomedica, Vienna, Austria) diluted 1:30. After washing two times 5min with cold PBS, sections were incubated with the secondary goat anti-rabbit IgG-Alexa 488 antibody (Invitrogen, Carlsbad, CA, USA) at a concentration of 20μg/ml.

#### 2.1.4 Proximity ligation assay

PLA (Duolink^®^, Sigma) was performed according to the manufacturer’s instructions. Briefly, in order to reduce autofluorescence, sections were immunofixed in acetone for 10min at -20°C. After rehydration with PBS at RT, Duolink Blocking Solution was applied for 1h at 37°C. The same primary antibodies as used for immunofluorescence (see above) were diluted in Duolink Antibody Diluent and applied for 1h at RT. After incubation with the PLUS and MINUS secondary antibodies conjugated with oligonucleotides delivered with the kit, the ligation solution containing ligase and oligonucleotides was added. During ligation over 30min at 37°C, the hybridizing connector oligonucleotides join the two PLA probes to a closed loop wherever they are in sufficient close proximity of <40nm. During amplification for 100min at 37°C, the PLA probe acts as a primer for the rolling-circle amplification. A polymerase generates a concatemeric product to which fluorescently labeled oligonucleotides hybridize, which amplifies the signal. Thus, the single wavelength PLA signal from the fluorescently labeled oligonucleotide originates only from sites where antibodies were in sufficiently close proximity. The control experiments were performed under identical conditions by using isotype control stains and primary and/or secondary antibodies only.

### 2.2 Microscopy

Images of trichrome-stained tissues were acquired using a Nikon Ti2 widefield microscope with 4x Plan Apo NA 0.2 or 20x Plan Apo NA 0.75 objectives (Nikon, Tokyo, Japan). Images were prepared using OMERO software. Immunofluorescence images of IF- and PLA-stained sections were acquired using a Nikon Ti2- Crest V3 (spinning disk) confocal microscope with a Nikon Plan Apo 60x 1.2 NA water immersion objective at 1.5 zoom, yielding a 0.07×0.07µm pixel size with the Photometrics Kinetix camera. Fluorescent single-bandpass emission filters with peak transmission and a bandwidth of 595/31 for Cy3 (in PLA), 685/40 for Cy5 (vWF respectively C3c), 511/20 for GFP (C4d), and multiband filter penta EM Multiband Penta CELESTA-DA/FI/TR/Cy5/Cy7-A (FF01-441/511/593/684/817) for Cy7 (C1q) were used. Bright-field images with and without polarized filter were acquired. Nikon NIS AR (version 5.3) was used as acquisition software. The excitation wavelength was 546nm (Cy3), 638nm (Cy5), 477nm (GFP), and 749nm (Cy7), respectively. Settings were chosen to avoid oversaturation in any of the samples and kept constant across acquisition.

### 2.3 Image analysis

Image analysis was performed using ImageJ software. Data analysis focused on manually drawn ROIs, outlining the intima, including endothelial cells and subendothelial space up to the internal elastic membrane, as visualized in a bright-field image. Images of diseased samples were categorized into regions of atheroma, atheroma with presence of CCs, atheroma with the presence of foam cells, or fibrotic regions. Categorization was performed according to a pathologist’s judgement of a serial trichrome-stained section. The presence of CCs was judged by the signal in the in polarized channel or the presence of typical crystal clefts occurring after washout during material processing. A healthy specimen contained only one region category: healthy intima. Each category (in diseased samples up to four categories; in healthy one category only) was sampled depending on the abundance of the manifestation with 4-9 images per region category, taking care that no overrepresentation of a single sample occurred. An analysis comparing luminal regions was performed and restricted to HA and DA specimens as anatomic structures were not well preserved in the TE samples, and estimation of the luminal side was not unambiguously identifiable.

For IF images, the mean ROI signal intensity was calculated. In addition, overlap values between the C1q and vWF signals were obtained by calculating Manders’ overlap coefficients using the JACoP plugin within Fiji software (22). Briefly, images were thresholded using the 99.5^th^ percentile of isotype control image signals, and the fraction of positive signals in the vWF channel overlapping positive signals in the C1q channel was calculated. PLA images were analyzed according to area with PLA-positive signal, as defined by a signal larger than the 99^th^ percentile of the isotype control signal.

### 2.4 Statistics

Statistical analyses were performed using Rstudio software version 2023.03.0 + 386. Data are expressed as mean ± S.D. To test for differences between groups, a non-parametric Kruskal–Wallis test was used when normal distribution was violated. *Post hoc* comparison was performed using a two-sided Mann–Whitney test with Bonferroni correction for multiple pairwise comparisons. Statistical significance was considered as * p ≤ 0.05, ** p<0.01, and ***p<0.001, respectively. IF signals were correlated using Spearman’s rank correlation coefficient.

## 3 Results

### 3.1 C1q and vWF are present in human carotid arteries

**Figure 1:**
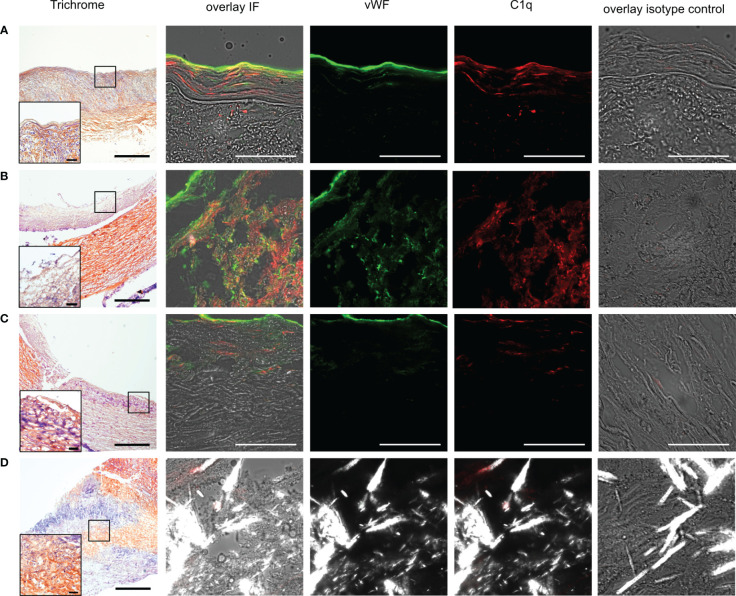
Presence of vWF and C1q in human carotid arteries. **(A)** Example images from frozen human carotid artery sections of autopsy material without atherosclerotic changes (healthy autopsy HA) and **(B–D)** specimen with manifestations of atherosclerosis, namely, **(B)** foam cells, **(C)** fibrotic changes, and **(D)** CCs. Data from B and C from different parts of the same specimen. First column shows trichrome staining in 4x resolution (scale bar=500µm), with insets at marked areas at 20x resolution (scale bar=50µm) for determination of morphology. The second column shows IF staining in corresponding specimen superimposed vWF (green), C1q (red) on brightfield images, and polarized filter acquisition for visualization of CCs. Third column demonstrates vWF channel, fourth column C1q channel, and fifth column isotype control. All IF images recorded at 60x magnification; scale bar=50µm.

Immunofluorescence staining was used to detect C1q and vWF in carotid arteries from autopsied patients without microscopic signs of atherosclerosis (healthy autopsy, HA), with macroscopic manifestation of atherosclerosis (diseased autopsy, DA), and with material from thromboendarterectomy procedures (diseased thromboendarterectomy, TE). In HA specimens, the vWF stain was most prominently localized luminally, corresponding to the endothelial lining, and in the subendothelial space of the intima, while C1q staining was more distributed (**Figure 1A**). Diseased samples contained various stages of atherosclerosis, varying from early intima fibrosis to late-stage atheroma with or without foam cells and CCs (**Figures 1B–D**). In diseased specimens, C1q signal intensity was distributed in a spotty fashion, with areas of high intensity and areas mainly devoid of C1q being present within the same specimen (compare **Figures 1B, C**). VWF was localized to the luminal endothelium and additionally showed a spotty distribution in deeper regions.

### 3.2 Higher signals of C1q and vWF in healthy material

**Figure 2:**
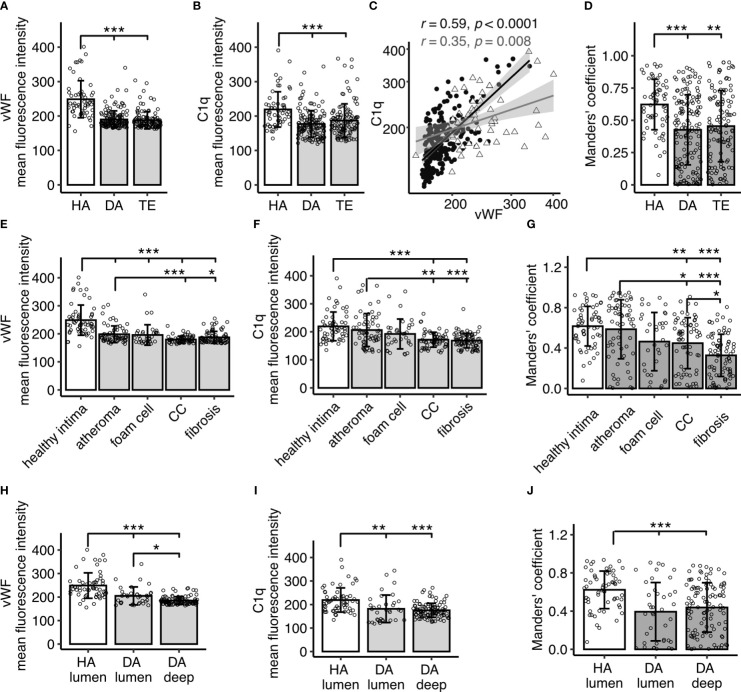
Quantitative analysis of IF signal for vWF and C1q in human carotid arteries and overlap calculated as Manders’ co-efficient. **(A)** Fluorescent signals for vWF and **(B)** C1q stain in specimen without atherosclerotic changes (healthy autopsy material, HA, n=7), material with atherosclerotic manifestation (diseased autopsy material, DA, n=7), and material from thrombendarteriectomy (TE, n=7), each dot representing a mean fluorescence signal from one 60x resolution image. **(C)** Spearman’s correlation analysis of vWF versus C1q fluorescence intensities, each datapoint representing a value from a single image. Filled black circle: diseased (DA and TE); open grey triangle: healthy (HA). **(D)** Manders’ co-efficient for HA, DA, and TE. **(E, F)** show IF signal analyzed according to images in different disease manifestation for vWF **(E)** and C1q **(F)**, respectively. **(G)** Manders’ co-efficient according to **(E, F)**. **(H, I)** show analysis focused on images with luminal content encompassing endothelium in HA and DA specimen versus deeper recording sites without lumen (DA deep) for vWF **(H)** and C1q signals **(I)**, respectively. **(J)** Manders’ co-efficient according to **(H, I)**. All data given as mean± S.D. p-values * <0.05, ** <0.01, ***<0.001.

Quantitative analyses of fluorescent signal intensities showed significant differences of vWF and C1q signal intensities between the three investigated groups (p<0.001 each), with significantly higher levels occurring in HA compared to DA (vWF p<0.001, C1q p<0.001) and TE (vWF p<0.001, C1q p<0.001) (**Figures 2A, B**). There was no significant difference between the diseased material from DA (autopsy) and TE (interventional) material, nor was there for vWF or C1q (p>0.05), thus making effects due to sample generation unlikely, e.g., intense contact with the bloodstream and coagulation factors during TE. In addition, we did not observe differences between symptomatic (TE) and asymptomatic atherosclerosis (DA). However, the correlation of vWF and C1q signal intensities was stronger in diseased samples (DA and TE) (r=0.59 p<0.001) than in healthy samples (r= 0.35, p=0.008) (**Figure 2C**). Signal intensities of isotype controls did not differ (data not shown).

Notably, signal intensities in diseased samples were less homogenously distributed than in healthy samples, leading to a higher variability. In order to investigate whether signal intensity in diseased samples was related to a particular manifestation of atherosclerosis, images were categorized into areas of atheroma, atheroma with presence of CCs, atheroma with foam cells, or fibrotic regions. Regions with the highest vWF and C1q content were atheroma areas, including those containing foam cells (**Figures 2E, F**). Signals for both C1q and vWF were significantly lower in areas with fibrotic lesions and CCs when compared to atheroma areas (C1q: atheroma vs CC p<0.01, atheroma vs fibrosis p<0.001; vWF atheroma vs CC p<0.001, atheroma vs fibrosis p<0.05).

VWF is known to be produced in the endothelium. To exclude effects due to sampling bias, i.e., all HA images encompassed endothelial lining, while a substantial number of images in DA samples were collected distant from the lumen within the plaque, an additional analysis segregating images from DA samples into lumen-containing versus deep tissue images was performed. The luminal DA vWF (**Figure 2H**, p<0.001) and C1q signal (**Figure 2I** p<0.01) significantly differed from HA, illustrating truly diminished signals of C1q and vWF in atherosclerosis.

### 3.3 Using Manders’ co-efficient to evaluate co-occurrence of C1q and vWF IF signal

Next, we addressed the question of C1q and vWF signal overlap. The Manders’ coefficient, demonstrating a co-occurrence of vWF and C1q, matched overall the above described finding for the individual IF signals, with highest overlap in the healthy samples as compared to the diseased samples (HA vs DA p<0.001, HA vs TE p<0.01, **Figure 2D**)) and higher overlap in atheroma and atheroma with foam cells (**Figure 2G**). The Manders’ coefficient differed significantly between HA and DA luminal regions and deep regions (both p<0.001), with the highest Manders’ coefficient in the HA luminal regions (**Figure 2J**). Notably, the variation in diseased samples was more pronounced.

### 3.4 Co-localization evaluated by using PLA

**Figure 3:**
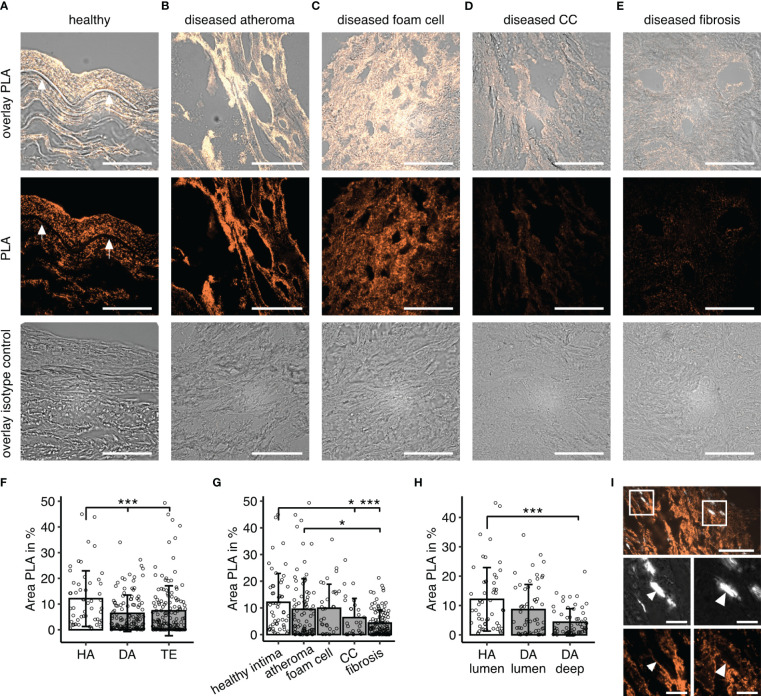
C1q-vWF co-localization determined by proximity ligation assay (PLA). **(A–E)** Example PLA staining visualizing C1q- vWF proximity in **(A)** healthy specimen, **(B)** diseased material in a region of atheroma, **(C)** foam cell, **(D)** CCs, and **(E)** fibrotic lesion; first row overlays PLA and brightfield, second row PLA only, and third row isotype control. All images in 60x magnification; scale bar=50µm. Arrows in **(A)** indicate the lamina propria. **(F)** Quantitative analyses of PLA fluorescent signals for C1q-vWF stain in specimen without atherosclerotic changes (healthy autopsy material, HA, n=7), material with atherosclerotic manifestation (diseased autopsy material, DA, n=7), and material from thrombendarteriectomy (TE, n=7), each dot representing mean fluorescent signal from one 60x resolution image. **(G)** PLA signal analyzed according to different disease manifestations. **(H)** Analysis focused on images with luminal content encompassing endothelium in HA and DA specimen versus deeper recording sites without lumen (DA deep). All data given as mean± S.D. P-values * <0.05, ***<0.001. **(I)** Example image of PLA in crystal regions with insets enlarged in lower panel. Surface of birefringent CCs seen in polarization filter channel marked with arrows. Scale bar in upper panel corresponds to 50µm and lower zoomed-in insets scale bar to 10µm.

To demonstrate that the co-occurrence corresponded to a meaningful binding of the two proteins, we performed PLA (**Figure 3**). Similar to IF data, we observed inhomogeneous signal distribution in diseased samples (**Figures 3A–D**) and PLA area -reflecting co-localization of vWF and C1q-was overall higher in healthy samples (HA vs DA and HA vs TE p< 0.001) (**Figure 3F**). With respect to disease manifestation, the area was lowest in fibrotic lesions of diseased samples, while values with very high signal coverage were seen in some regions with foam cells and atheroma (**Figure 3G**). PLA signal of HA specimens differed from deep DA (p< 0.001), while luminal DA regions only expressed a statistical trend (p=0.07) (**Figure 3H**).

### 3.5 Co-localization on CCs

Based on previous *in vitro* experiments, our assumption was that co-occurrence would occur at the interface of the CCs (18). However, PLA signal was low overall in images containing CCs. Nevertheless, there were clear instances with labeling of CCs (**Figure 3I**), and this observation may be underestimated as for this analysis tissues were immunofixed in acetone in order to reduce auto-fluorescence occurring in the wavelength of the PLA probe. This procedure might have caused the reduction of CC load by washout (8). Thus, though not being a prominent feature, C1q-vWF complexes occur at the outline of CCs in human atherosclerotic lesions.

### 3.6 Complement activation

To evaluate a possible impact of vWF binding to C1q on downstream complement activation, a subset of samples was stained for the presence of complement C3c and C4d (**Supplementary Figure 1**) We used 20x resolution images to compare staining across consecutive sections. Distributions of each of the named molecules were non-uniform. While in some areas of the samples, the overall distribution of downstream complement products seemed to resemble the PLA signal for co-localized C1q-vWF (exemplified in **Supplementary Figure 1E**, foam cell area), there were also areas predominantly devoid of a PLA signal nevertheless showing a C3c signal. The highest C4d signals were observed in atheroma areas with necrotic appearance.

## 4 Discussion

Here, we demonstrate the presence and co-localization of C1q and vWF in the intima of human carotid arteries, which is occurring predominantly in healthy tissue and is reduced in atherosclerosis.

VWF localized to the endothelium and the subendothelial space, in agreement with its primary production in tissue by endothelial cells, and was reduced in diseased material even when limiting the analysis to luminal regions (**Figure 2H**). C1q staining was similarly reduced in diseased material, albeit expressing a higher degree of variability than the vWF signal. This may reflect the more diverse source of tissue C1q deriving from dendritic cells, macrophages, and the blood stream (23). Compared to healthy arteries, staining was spotty for C1q and vWF in diseased samples, with the regions of highest vWF and C1q signals in the atheroma and foam cell areas being well in line with previous reports on the vWF localization in fatty streaks (24) and the macrophages as the origin of foam cells (25).

Furthermore, we demonstrated the presence of C1q-vWF complexes by PLA occurring predominantly in healthy material and reduced in atherosclerotic tissue. While signals were comparable between the healthy intima and atheroma and regions encompassing foam cells, lower values were seen in fibrotic lesions and areas with CCs.

As the current study is observational, we may only speculate on the functional relevance of our findings. In principle, there are three possible interpretations: First, the reduced C1q-vWF interaction in atherosclerosis could be a non-causal observation. However, it is striking that the highest co-localization occurred in the subendothelial space of healthy material, a space known to be the location of initiation of retention of lipoproteins and, by this, the initiation zone for atherosclerosis (26).

Second, it is possible that reduced levels of C1q and C1q-vWF co-occurrence reflect an adaptive downregulation in diseased atherosclerotic tissue, e.g., to prevent thrombus formation, as C1q-vWF can induce platelet rolling and adhesion (19). Moreover, the deficiency of each protein individually has been shown to be atheroprotective in animal studies (13, 27), and C1q is expected to have prothrombotic features based on the observation of prolonged bleeding time in C1qKO mice (28).

Conversely, a third interpretation is that C1q-vWF has protective effects that are lost during disease progression. These effects could be anti-inflammatory. While it is not known whether C1q-vWF has anti-inflammatory properties *per se*, there is evidence for C1q-vWF bound to CCs (CC-C1q-vWF complexes) to have a rather anti-inflammatory effect on macrophages, reducing IL-1 release (18). However, although we demonstrate the presence of C1q-vWF in proximity to CCs (**Figure 3I**), it was not a predominant phenomenon. C1q deposition was rather observed in the surrounding tissue of CC than being localized on CCs themselves, in line with data shown by others (2). CCs are partially removed during tissue processing (8), which becomes particularly apparent in the needle-shaped CC clefts, but might be missed in smaller and differential appearance of CCs present in the atheroma core. Thus, it is likely that the co-occurrence of C1q-vWF in relation to CCs is underestimated in the presented study.

Alternatively, protective effects of C1q-vWF could potentially also be related to an attenuation of downstream classical pathway complement activation, which has been associated with the progression of atherosclerosis (3, 4, 9, 10). Indeed, the most well-known endogenous inhibition via complement inhibitors points toward a net increase in complement activation in plaques (29). We performed C3c and C4d stains in consecutive slides and observed high levels in atheroma areas. However, there was no obvious inverse relation between the co-localization of C1q and vWF and the presence of downstream complement activation, e.g., less complement activation in areas with higher abundance of interaction of C1q and vWF. It is important to note that C4d and C3c could potentially derive from activation by another pathway of the complement cascade rather than the classical pathway, i.e., C3 activation via all pathways and C4 via the lectin pathway in addition to the classical pathway. The net effect of complement activation in atherosclerosis appears to be regulated independently of C1q-vWF interaction. The exploration of potentially locally relevant effects of vWF on classical pathway activation will require more detailed studies.

### 4.1 Conclusion

This study describing C1q and vWF deposition in atherosclerosis provides further insights into the steadily growing evidence of a cross-talk between complement and hemostasis. Based on our previous *in vitro* finding of the formation of CC-C1q-vWF complexes leading to downstream anti-inflammatory effects on human macrophages, we can now show that C1q-vWF interaction also occurs *ex vivo*. Although this interaction can also be demonstrated in areas with CCs, it is more abundant in healthy intima. Future studies will be required to explore the exact function and relevance of this unexpected finding.

## Data availability statement

The raw data supporting the conclusions of this article will be made available by the authors, without undue reservation.

## Ethics statement

Collection and use of patient tissue were approved by the local Ethical Committee of Northwestern and Central Switzerland (EKNZ No. 2019-01490). The studies were conducted in accordance with the local legislation and institutional requirements. The participants provided their written informed consent to participate in this study.

## Author contributions

KS: Conceptualization, Data curation, Formal analysis, Investigation, Methodology, Visualization, Writing – original draft, Writing – review & editing. CD: Conceptualization, Formal analysis, Investigation, Methodology, Visualization, Writing – original draft, Writing – review & editing. MP: Writing – review & editing. KG: Formal analysis, Resources, Writing – review & editing. BK: Resources, Writing – review & editing. MT: Conceptualization, Funding acquisition, Project administration, Resources, Supervision, Writing – review & editing.
