# Supplementary material for: Complement C1q and von Willebrand factor interaction in atherosclerosis of human carotid artery
Source: Front Immunol. 2023 Dec 14;14:1265387. doi: 10.3389/fimmu.2023.1265387 (PMC10753016; doi:10.3389/fimmu.2023.1265387)
Supplement: Supplementary file 1 [file DataSheet_1.pdf]

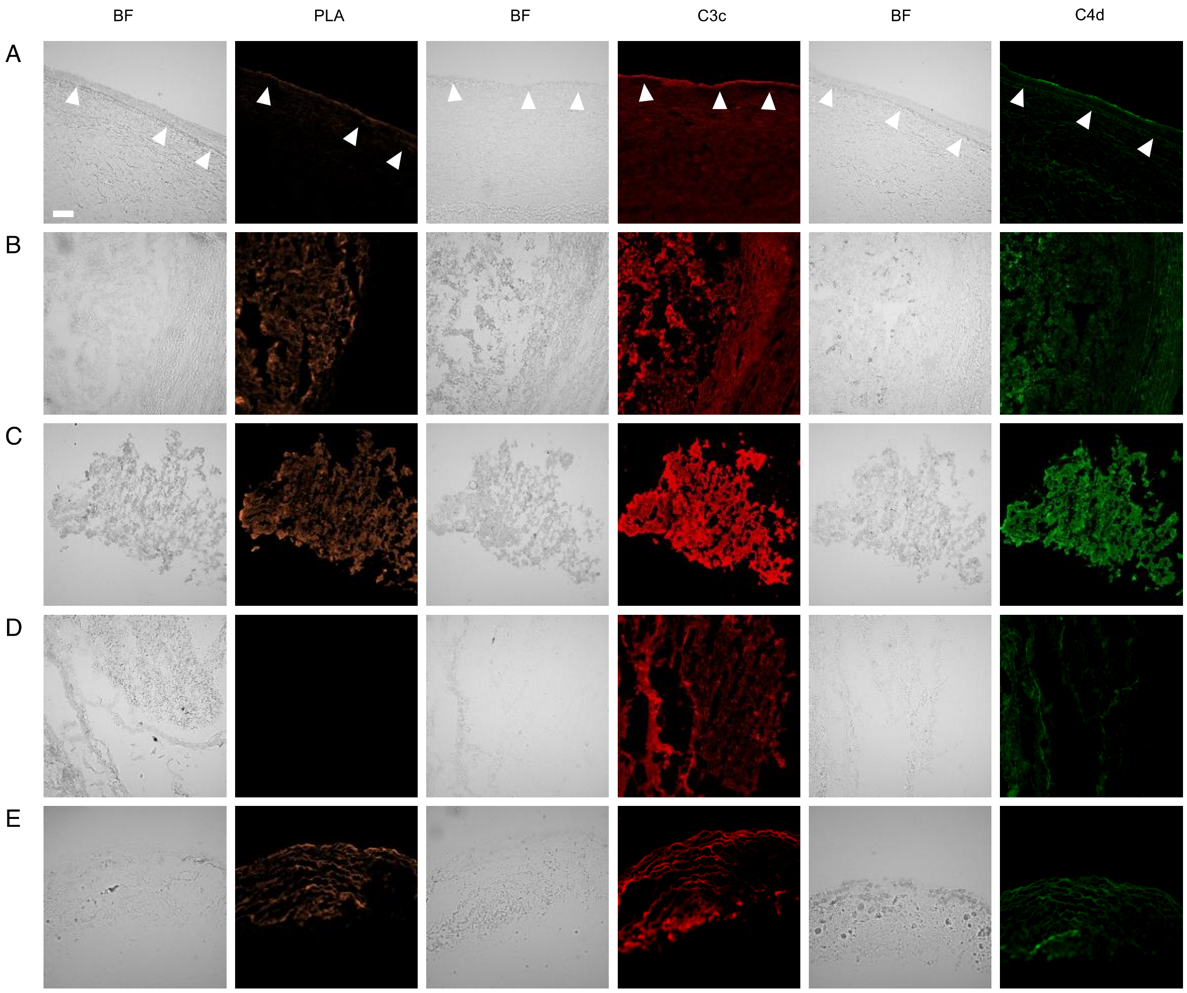

Supplementary Figure 1: Comparison of PLA Signal Reflecting vWF and C1q Co-localisation (orange) to Complement Split Products C3c (red) and C4d (green) in Serial Sections of Human Carotid Arteries. (A) Example images from frozen human carotid artery sections of autopsy material without atherosclerotic changes (healthy autopsy HA) and **(B- E)** specimen with manifestations of atherosclerosis namely **(B-C)** atheroma and **(D-E)** foam cell area. First, third and fifth column shows brightfield (BF) images with corresponding IF image in following column in 20x resolution (scale bar=100µm, arrows indicate the lamina propria as boarder of the intima).
